# Professional Academies: The Duty to Lead

**DOI:** 10.1111/1751-7915.70388

**Published:** 2026-06-07

**Authors:** Kenneth Timmis, Paul Rainey, Purificación Lopez‐García, Ines Mandić‐Mulec, Stipan Jonjić, Roberto Kolter, Anna Karnkowska, Chantal Abergel, Pablo Ivan Nikel, Juan Luis Ramos

**Affiliations:** ^1^ Institute for Microbiology Technical University of Braunschweig Braunschweig Germany; ^2^ Department of Microbial Population Biology Max Planck Institute for Evolutionary Biology Plön Germany; ^3^ Laboratory of Biophysics and Evolution, CBI, ESPCI Paris Université PSL, CNRS Paris France; ^4^ Ecologie Systématique Evolution CNRS, Université Paris‐Saclay Gif‐sur‐Yvette France; ^5^ Department of Microbiology University of Ljubljana Ljubljana Slovenia; ^6^ Center for Proteomics, Faculty of Medicine University of Rijeka Rijeka Croatia; ^7^ Department of Microbiology Harvard Medical School Boston Massachusetts USA; ^8^ Institute of Evolutionary Biology, Faculty of Biology, Biological and Chemical Research Centre University of Warsaw Warsaw Poland; ^9^ Information Génomique & Structurale CNRS & Aix‐Marseille Université Marseille France; ^10^ Systems Environmental Microbiology Group, The Novo Nordisk Foundation Center for Biosustainability Technical University of Denmark Lyngby Denmark; ^11^ Consejo Superior de Investigaciones Cientificas Estación Experimental del Zaidín Granada Spain

## Abstract

Scientific academies are not prize clubs. They are public institutions with independence, expertise, and convening power. In a period of climate instability, widening inequality, and declining trust in science, these attributes carry a duty to lead. This is not leadership as authority or control, but as stewardship: setting standards, shaping policy with evidence, mobilising networks to deliver practical solutions, and assessing impact in the public interest. Academies exist because of public investment in their members; their obligation is to apply collective expertise to the most pressing and serious societal challenges of the time.

Election to a scientific academy is a distinction that recognises significant achievement. For most new members it brings satisfaction, opportunity to engage with peers, participation in committees, and influence over scientific priorities. For academies, it renews and widens expertise, and strengthens capacity to promote excellence (Halpern 1997).

Some academies and some of their members may also drift toward subconscious self‐congratulation or internal preoccupation (Franck 1999). However, recognition is not an end in itself and prestige should not detach from responsibility. The education systems, research infrastructures, and public resources that enable success come from society at large. Membership therefore entails a duty to return value to society.

Academies represent concentrations of talent and knowledge, resources to which society may turn when required. Their members advance research and education within their disciplines, advances that may have important applications which can benefit humankind. But science and society do not progress in parallel. Politics, economics, inequality, and environmental degradation often obstruct the translation of scientific progress into social benefit (https://sdgs.un.org/2030agenda; Anand et al. 2023; Timmis et al. 2025). Members of academies are also citizens with the capacity to apply their expertise to broader societal issues (Timmis and Hallsworth 2024). Academies make available their resources to society and the body politic but should not develop their own political agenda; they must act as a “conscience of society,” encouraging dialogue, tolerance, and a value system that strengthens communities.

Key areas of action of scientific academies include advancing disciplinary excellence and innovation, promoting the development and mentoring of younger and especially disadvantaged scientists, transferring technologies to regions where they are needed, and developing low‐cost or “frugal” innovations to widen access. Where possible, they should also address urgent planetary problems such as climate change (Nguyen and Casadevall 2021; Crowther et al. 2024; https://asm.org/getmedia/b72d7f82‐8fe4‐4f49‐867c‐04464a654886/Microbial‐Solutions‐for‐Climate‐Change‐Toward‐an‐Economically‐Resilient‐Future.pdf), desertification (Timmis and Ramos 2021), ecosystem degradation, and biodiversity loss, as well as promote education as a cornerstone of social resilience (Timmis 2023; Benavot et al. 2024; Timmis et al. 2024; https://www.globalpartnership.org/blog/kofi‐annan‐knew‐importance‐education).

The channelling of scientific knowledge into policy decisions requires engagement with policymakers. Some academies already have access to policymakers (Halpern 1997; Quéré 2010; Timmis and Hallsworth 2024; https://www.un.org/technologybank/academies‐of‐sciences), and their members, through both expertise and civic engagement, help shape evidence‐based policy. Examples include the Royal Society (https://royalsociety.org/news‐resources/publications/2025/spending‐review‐phase‐2/?utm_source=chatgpt.com; https://royalsociety.org/news/2023/01/privacy‐enhancing‐technologies/?utm_source=chatgpt.com), the National Academies of Science, Engineering and Medicine (https://nap.nationalacademies.org/topic/423/policy‐for‐science‐and‐technology), and the American Academy of Microbiology (https://asm.org/academy/climate‐change‐and‐microbes‐scientific‐portfolio), which have established science‐policy interfaces and advisory roles. Such engagement demonstrates the potential of academies to influence education, research and public policy.

Like other institutions, academies follow the Pareto Principle (Pareto 1896–1897): a minority of members account for most activity (Timmis et al. 2023). This balance is unsurprising given the competing demands on active scientists. However, academies are also home to retired members that encompass an experienced group with fewer professional obligations. This “silver dividend” represents a valuable, often underused resource (https://www.adb.org/sites/default/files/publication/864241/ewp‐678‐population‐aging‐silver‐dividend‐economic‐growth.pdf). With academy leadership and vision, retired members can help drive academy initiatives and mentor younger colleagues.

An academy that mobilises its membership toward shared goals can have disproportionate impacts (Halpern 1997; Timmis and Hallsworth 2024); visible and sustained engagement from academies can encourage other organisations to follow, amplifying benefits across society.

Periods of crisis and inequality call for leadership rooted in knowledge and integrity (Anand et al. 2023; Timmis et al. 2025). Academies are positioned to provide such leadership. Their members, recognised for exceptional achievement, bear a special responsibility to apply their abilities to public good. In so doing, they reaffirm the social contract that underpins their existence. The creation of academies is justified not only by the pursuit of knowledge but also by their capacity to lead on issues vital to humanity.

## Author Contributions

**Paul Rainey:** conceptualization, writing – original draft, writing – review and editing. **Anna Karnkowska:** writing – review and editing. **Pablo Ivan Nikel:** writing – review and editing. **Kenneth Timmis:** conceptualization, writing – original draft. **Roberto Kolter:** writing – review and editing. **Stipan Jonjić:** writing – review and editing. **Chantal Abergel:** writing – review and editing. **Ines Mandić‐Mulec:** writing – review and editing. **Juan Luis Ramos:** writing – review and editing. **Purificación Lopez‐García:** writing – review and editing.

## Funding

The authors have nothing to report.

## Conflicts of Interest

The authors declare no conflicts of interest.

## Data Availability

Data sharing not applicable as no datasets were generated or analysed.
